# Effect of long working hours and insomnia on depressive symptoms among employees of Chinese internet companies

**DOI:** 10.1186/s12889-021-11454-9

**Published:** 2021-07-16

**Authors:** Xiaoman Liu, Chao Wang, Jin Wang, Yuqing Ji, Shuang Li

**Affiliations:** 1 National Institute of Occupational Health and Poison Control, Chinese Center for Disease Control and Prevention, 29 Nanwei Road, Xicheng District, Beijing, P.R. China 100050; 2 Department of Laboratory Science and Technology & Vaccine Research Center, School of Public Health, Peking University, No.38 Xueyuan Road, Haidian District, Beijing, P.R. China 100191

**Keywords:** Long working hours, Insomnia, Depressive symptom, Interaction effect

## Abstract

**Background:**

In China, long working hours and insomnia are relatively common among internet company employees. Considering that both can affect mental health, we examined their independent and interaction effects on these employees’ depressive symptoms (DS).

**Methods:**

We analyzed data from the 2016 occupational health questionnaire survey conducted in 35 large-, medium-, and small-scale internet companies. Overall, 3589 full-time employees were recruited to evaluate the association among working hours, insomnia, and DS. The Patient Health Questionnaire (PHQ-9) was used to assess DS. The association of DS (PHQ-9 ≥ 10) with working hours (≤40, 41–50, 51–60, and > 60 h/week), insomnia (with or without), and interaction of both was estimated using multivariable logistic regression analysis.

**Results:**

Compared with the group working for ≤40 h/week, the adjusted odds ratios (ORs) for DS among participants who worked for 41–50 h/week, 51–60 h/week, and > 60 h/week were 1.32 (1.11–1.56), 1.74 (1.35–2.24), and 2.54 (1.90–3.39), respectively. The ORs for DS among those with insomnia were 2.36 (2.04–2.74) after adjusting for general characteristics. The ORs for DS related to insomnia were similar [1.91 (1.46–2.50), 2.00 (1.61–2.50), respectively] in the participants who worked for < 50 h/week. However, among participants working for 51–60 h or > 60 h/week, the adjusted ORs for DS substantially increased to 4.62 (2.90–7.37) and 5.60 (3.36–9.33), respectively. Moreover, among the participants with insomnia, working overtime showed a greater association with DS.

**Conclusions:**

We showed that long working hours and insomnia are independent factors associated with the prevalence of DS; furthermore, an interaction effect of long working hours and insomnia on DS was observed. For relieving DS in internet company employees, it is important to reduce insomnia.

**Supplementary Information:**

The online version contains supplementary material available at 10.1186/s12889-021-11454-9.

## Background

Recently, the internet industry in China has undergone an explosive increase in discussions regarding the “996” work system (working hours: 9 am to 9 pm, 6 days per week). In 2019, for the first time, a large-scale universal discussion was conducted among Chinese internet company employees regarding overtime, overwork, and enterprise violation of labor contracts, and this discussion has had an international impact. The survival of the internet industry relies on continuous innovation, which is considered a matter of concern by these companies, thereby rendering long working hours a commonplace in Chinese internet companies [1, 2]. However, extending working hours does not necessarily improve the efficiency and ability of employees; on the contrary, employees have little time to spend with and care for their family, which can lead to poorer work quality and declining work ability as well as a result in damage to their physical and mental health [3–5]. In recent years, European countries have been the focus of research on the adverse effects of long working hours, followed by East Asia, particularly Japan and South Korea. Research conducted in China on long working hours is limited; however, the working hours followed in China are among the longest in the world, overtime, overwork and even “karoshi” (death from overwork) are common among Chinese individuals [6]. Therefore, further attention is required regarding the adverse effects caused by long working hours in China.

Depression is one of the most common mental health disorders. It is one of the leading causes of disability worldwide and a major contributor to the overall global burden of disease [7]. In China, the lifetime prevalence of depression is approximately 6% [8], and it is the fourth leading cause of disability [9]. Several factors contribute to depressive symptoms (DS); numerous studies have shown that working long hours is likely an important risk factor for DS [10–14]. A systematic review and meta-analysis involving approximately 190,000 participants from 28 prospective cohort studies in 35 countries reported that an increased risk of depression was linked to prior overtime work in Europe and, to a greater extent, in Asia [15]. The mechanisms linking long working hours and DS involved social support, high demand and low control, effort–reward imbalance, effort–recovery imbalance, and others [16–18]. Long working hours lead to sleep deprivation and lack of recovery from work-related stress, thereby reducing non-working time, increasing injuries, and prolonging exposure to work needs and workplace hazards [19]. These direct consequences might develop into depression by fatigue; negative emotions owing to lack of time for socialization and life; and bad feelings toward work because of increased exposure to workplace demands and hazards [20].

Considering that long working hours are positively associated with insomnia, few studies have been conducted to illustrate the effects of their interaction on subsequent health issues, such as depressive disorder, because both interfere with the energy recovery and mental burnout of individuals. This hypothesis has been indicated by Nakata, who reported that the combination of long working hours and short sleep periods posed the strongest risk for DS among full-time employees [21]. Regarding associations between insomnia and depression, causation, i.e., the interacting effects of insomnia and working time on mental health, remain poorly understood. Insomnia is one of the most common sleep disorders. Studies have shown that approximately 70% of patients with depression have insomnia [22], and patients with insomnia without DS have a two-fold risk of developing depression compared with individuals without insomnia [23]. According to an epidemiological study, the prevalence rate of insomnia in Chinese adults was between 9.2 and 11.2% [24]. In 2016, the first survey on the sleep quality of Chinese internet company employees had found that approximately 80% of the individuals experienced poor sleep quality, 71.3% experienced insomnia, and 60% often worked overtime [25].

Therefore, the objective of the present study was to examine the effect of long working hours and insomnia on the DS of full-time employees using data from 35 large-, medium-, and small-scale internet companies in China. We hypothesized that long working hours and insomnia are both independent factors associated with the prevalence of DS among internet company employees and that there may be an interaction effect of long working hours and insomnia on DS.

## Methods

### Study design and population

The study used a cross-sectional design. From June to September 2016, employees from 35 large-, medium-, and small-scale internet companies were selected from Beijing, Shandong, and Zhejiang provinces. The enterprises selected were based on the broad internet enterprise classification, including basic, service, and terminal layer internet enterprises. In each enterprise, the anonymous self-administered questionnaire survey was employed, and it was distributed, guided, completed, and reviewed by the investigators after unified training. Auditors were assigned to review issues related to quality, such as missing items and logical contradictions. A total of 4355 participants with various job positions participated in the survey. The inclusion criteria for participants were as follows: employees who had worked in their current position for at least 6 months and who did not have any recent mental disease or are not on any psychotropic medication. Finally, 3589 qualified questionnaires were received, with an effective recovery rate of 82.41%. The study was approved by the Medical Ethics Committee of the National Institute of Occupational Health and Poison Control. Each participant provided written informed consent.

### DS

DS was measured using a Chinese version of the Patient Health Questionnaire (PHQ-9) [26]. The 9-item DS scale measures the frequency levels of DS experienced in the past 2 weeks. The 4 frequency levels are 0 (never), 1 (occasionally), 2 (more than half), and 3 (always). The PHQ-9 scale cut-off score is 10, which differentiates between those exhibiting high levels of DS (score ≥ 10) and those with lower levels of such symptoms (score < 10). PHQ-9 is a simple and effective instrument for making diagnoses and assessing the severity of depressive disorders, particularly in busy clinical practice settings [27].

### Number of working hours

Working hours were determined by an open-ended question: for how many hours do you usually work in a week (including overtime)? According to the relevant provisions of the labor law and the regulations of the State Council on employees’ working hours, the current standard working hour system in China is 8 h/day and 40 h/week [28, 29]. Therefore, the number of working hours was grouped into 4 categories: ≤40, 41–50, 51–60, and > 60 h/week.

### Insomnia

Insomnia was determined using a Chinese version of the Sleep Questionnaire (Supplementary file 1), which was modified from Nakata’s Self-administrated Sleep Questionnaire [30]. The questionnaire included 3 questions about subjective sleep habits during the last 1 year. Difficulty initiating sleep (DIS) was defined as requiring > 30 min to fall asleep. Difficulty maintaining sleep (DMS) and early morning awakening (EMA) were defined by a response of “>3 times a week” to the second and third questions [31, 32]. The presence of insomnia was defined by at least 1 positive response to questions regarding DIS, DMS, or EMA.

### Covariates

Demographic, socioeconomic, and health-related covariates were included in the present study. The covariates were age (16–25, 26–30, 31–35, 36–40, or ≥ 41 years), sex (male or female), education level (high school, junior college, college, or graduate school), marital status (married, single, divorced, or widowed), income (¥) (≤4999, 5000–7999, 8000–11,999, or ≥ 12,000), exercise frequency (never, 1–3 times per month, 1–2 times per week or ≥ 3 times per week), dietary habits (regular, occasional, irregular, often irregular, or totally irregular), and smoking status (nonsmoker, former smoker, or current smoker). Exercise frequency was measured using the question: “Do you perform any exercise?” Exercising for > 30 min was recommended for effective health promotion [33].

### Statistical analysis

The chi-squared test was used to compare group differences in categorical data. The study hypotheses were analyzed based on logistic regression analysis to investigate the relationship among work time (WT), insomnia, and depression. The interaction effect of WT and insomnia on DS was investigated using stratified logistic regression analysis. For the analysis of the independent effects, WT and insomnia were first introduced into the regression simultaneously after adjusting for general characteristics, such as sex, age, education level, marital status, income, exercise frequency, dietary habits, and smoking status. The regression model then further introduced an interaction value of WT × insomnia to detect the interaction effect. The interaction value of WT in model 2 was introduced as a continuous variable. A *p*-value of < 0.05 was considered to indicate statistical significance. All analyses were performed using IBM Statistics SPSS 22.0.

## Results

### Characteristics of study participants

Overall, 4355 questionnaires were issued in the survey and 3589 (82.41%) completed questionnaires were eligible for the analysis. Among the entire sample, 56.3% of the participants were males, and the average age was 33 ± 9 years (range: 17–60 years). Further, 56.0% of the participants received a college education, with 9.6% having only a high school background. Most participants (65.8%) were married. Approximately 70% of the participants earned < ¥8000/month. The chi-squared test showed that except for sex, most of the distribution of general characteristics between the DS groups was statistically significant (*p* < 0.05) (Table 1). Participants who worked for **>** 60 h/week showed the highest rate of DS (48.3%); 41.7%were classified to have insomnia, among which 53.7% had DS (Table 1).

**Table 1:** General characteristics and depressive symptoms

Characteristics		Total N(%)	Depressive symptoms		
			Negative n(%)	Positive n(%)	***p***-value
**Total**		3589 (100.0)	2175 (60.6)	1414 (39.4)	
**Age group**	16–25	625 (17.4)	421 (67.4)	204 (32.6)	< 0.001
	26–30	954 (26.6)	665 (69.7)	289 (30.3)	
	31–35	753 (21.0)	478 (63.5)	275 (36.5)	
	36–40	507 (14.1)	275 (54.2)	232 (45.8)	
	≥41	750 (20.9)	387 (51.6)	363 (48.4)	
**Sex**	Male	2022 (56.3)	1267 (62.7)	755 (37.3)	0.371
	Female	1567 (43.7)	959 (61.2)	608 (38.8)	
**Education**	High school	344 (9.6)	209 (60.8)	135 (39.2)	< 0.001
	Junior college	521 (14.5)	280 (53.7)	241 (46.3)	
	College	2009 (56.0)	1242 (61.8)	767 (38.2)	
	Graduate school	715 (19.9)	495 (69.2)	220 (30.8)	
**Marital status**	Married	2363 (65.8)	1401 (59.3)	962 (40.7)	< 0.001
	Single	1159 (32.3)	794 (68.5)	365 (31.5)	
	Separated/divorced/widowed	67 (1.9)	31 (46.3)	36 (53.7)	
**Income(¥)**	≤4999	1319 (36.8)	717 (54.4)	602 (45.6)	< 0.001
	5000–7999	1105 (30.8)	690 (62.4)	415 (37.6)	
	8000–11,999	767 (21.4)	534 (69.6)	233 (30.4)	
	≥12,000	398 (11.1)	285 (71.6)	113 (28.4)	
**Exercise**	Never	527 (14.7)	278 (52.8)	249 (47.2)	< 0.001
	1–3 times/month	1626 (45.3)	1003 (61.7)	623 (38.3)	
	1–2 times/week	927 (25.8)	598 (64.5)	329 (35.5)	
	≥3 times /week	509 (14.2)	347 (68.2)	162 (31.8)	
**Dietary habits**	Regular	2069 (57.6)	1406 (68.0)	663 (32.0)	< 0.001
	Occasionally irregular	968 (27.0)	579 (59.8)	389 (40.2)	
	Often irregular	440 (12.3)	193 (43.9)	247 (56.1)	
	Totally irregular	112 (3.1)	48 (42.9)	64 (57.1)	
**Smoking status**	Nonsmoker	2701 (75.3)	1725 (63.9)	976 (36.1)	< 0.001
	Former smoker	287 (8.0)	153 (53.3)	134 (46.7)	
	Current smoker	601 (16.7)	348 (57.9)	253 (42.1)	
**Work time**	≤40 h/week	1147 (32.0)	752 (65.6)	395 (34.4)	< 0.001
	41–50 h/week	1641 (45.7)	982 (59.8)	659 (40.2)	
	51–60 h/week	420 (11.7)	244 (58. 1)	176 (41.9)	
	> 60 h/week	381 (10.6)	197 (51.7)	184 (48.3)	
**Insomnia**	Negative	2091 (58.3)	1481 (70.8)	610 (29.2)	< 0.001
	Positive	1498 (41.7)	694 (46.3)	804 (53.7)	

### Analyses among WT, insomnia, and depression

The majority of participants worked for ≤50 h/week, which accounted for 77.7% of the total participants. Those who worked for > 50 h/week reported a significantly higher rate of DS, particularly when they worked for > 60 h/week. Compared with the group working for ≤40 h/week, the odds ratios (ORs) for DS among those who worked for 41–50, 51–60, and > 60 h/week showed a gradual increase and adjusted ORs were 1.32 (1.11–1.56), 1.74 (1.35–2.24), and 2.54 (1.90–3.39), respectively. Similarly, 53.7% of the participants with insomnia experienced DS, which was a significantly higher proportion than that of participants without insomnia. Logistic regression showed that OR for DS among those with insomnia was 2.36 (2.04–2.74) after adjusting for general characteristics (Table 2). To test whether there is an interaction effect of WT and insomnia on DS, we further introduced an interaction value of WT × insomnia into the regression. The interaction coefficient was found to be 1.35 (1.15–1.59) with statistical significance (Table 2).

**Table 2:** Analyses among work time, insomnia, and depression

	Values to be analyzed	Depressive symptom		
		ORa	95%***CI***	***p***-value
**Model 1**	**WT**			
	≤40 h/week	–	–	–
	41–50 h/week	1.32	1.11–1.56	< 0.001
	51–60 h/week	1.74	1.35–2.24	< 0.001
	> 60 h/week	2.54	1.90–3.39	< 0.001
	**Insomnia**			
	Negative	–	–	
	Positive	2.36	2.04–2.74	< 0.001
**Model 2**	**WT × Insomnia**	1.35	1.15–1.59	< 0.001
	**Nagelkerke R2**	0.171		

*Note*: Model 1: work time and insomnia were simultaneously introduced into the regression adjusted by sex, age, education level, marital status, income, exercise frequency, dietary habits, and smoking status. Model 2 further introduced an interaction value of WT × insomnia based on Model 1; only the interaction coefficient is shown in Table 2. WT of interaction value in model 2 was introduced as a continuous variable. *WT* work time; a: *p* < 0.05; *CI* confident interval; *ORa* odds ratio adjusted by general characteristics such as sex, age, education level, marital status, income, exercise frequency, dietary habits, and smoking status

The interaction effect was then illustrated in two manners from different perspectives by showing the exact moderating effects of WT or insomnia on each other. We first tested the association of insomnia with DS within each category of WT through a stratified regression analysis, as shown in Table 3. The results show that the association of insomnia with DS relies on the number of hours for which employees work. Insomnia showed increasing ORs for DS as WT increased. The ORs for DS related to insomnia are similar [1.91 (1.46–2.50) and 2.00 (1.61–2.50), respectively] in the participants who worked for < 50 h/week. However, among the participants working for 51–60 or > 60 h/week, the adjusted ORs substantially increased to 4.62 (2.90–7.37) and 5.60 (3.36–9.33), respectively (Table 3).

**Table 3:** Effects of insomnia on depression moderated by work time

Insomnia	Depressive symptom			
	WT: ≤40 h	WT: 41–50 h	WT: 51–60 h	WT: > 60 h
	***ORa*** (95%***CI)***	***ORa*** (95%***CI)***	***ORa*** (95%***CI)***	***ORa*** (95%***CI)***
**Negative**	–	–	–	–
**Positive**	1.91 (1.46–2.50)	2.00 (1.61–2.50)	4.62 (2.90–7.37)	5.60 (3.36–9.33)
**Nagelkerke R2**	0.143	0.191	0.313	0.284

*Note*: *WT* work time; *ORa* odds ratio adjusted by sex, age, education level, marital status, income, exercise frequency, dietary habits, and smoking status. *CI* confidence interval

For assessing the association between WT and DS moderated by insomnia, another stratified regression analysis was conducted. The results indicated that for participants without insomnia, working overtime but for **<** 60 h/week showed a relatively weak association with DS [adjusted ORs: 1.30 (1.03–1.65) and 1.30 (0.91–1.86) for 41–50 and 51–60 h/week, respectively]. However, participants working for **>** 60 h/week showed a significant OR of 1.82 (1.23–2.69) compared with those working for ≤40 h/week (reference group). Conversely, among participants with insomnia, working overtime showed a greater influence on DS. The adjusted ORs for 41–50, 51–60, and > 60 h/week were 1.35 (1.06–1.73), 2.48 (1.70–3.62), and 4.16 (2.62–6.60), respectively, compared with the reference group of ≤40 h/week (Table 4).

**Table 4:** Effects of work time on depression moderated by insomnia

Work time	Depressive symptom	
	Insomnia negative	Insomnia positive
	***ORa*** (95%***CI)***	***ORa*** (95%***CI)***
**≤40 h/week**	–	–
**41–50 h/week**	1.30 (1.03–1.65)	1.35 (1.06–1.73)
**51–60 h/week**	1.30 (0.91–1.86)	2.48 (1.70–3.62)
**> 60 h/week**	1.82 (1.23–2.69)	4.16 (2.62–6.60)
**Nagelkerke R2**	0.133	0.104

*Note*: *WT* work time; *ORa* odds ratio adjusted by general characteristics as sex, age, education level, marital status, income, exercise frequency, dietary habits, and smoking status; *CI* confidence interval

## Discussion

The present study assessed the relationship among long working hours, insomnia, and DS. The results confirmed our hypotheses that long working hours and insomnia are both independent factors associated with the prevalence of DS among internet company employees. In addition, there exists an interaction effect of long working hours and insomnia on DS. Particularly, among those with insomnia, a dose–response relationship was observed between working hours and DS; the ORs for DS were more than double for those working for 51–60 h/week and more than four-fold for those working for > 60 h/week compared with those working for < 40 h/week. Among those without insomnia, the association between working hours and DS was more modest, with elevated odds of DS seen primarily among those working for > 60 h/week.

In the present study, working overtime was one of the predictors of DS. This finding was consistent with the systematic reviews and/or meta-analyses, which have reported a positive association between long working hours and depression [4, 34, 35]. Owing to working overtime, less time is available for social activities, leading to damage to interpersonal relationships, social exchange, and social support, which are suggested as beneficial factors for personal mental health [36]. As indicated by Kleppa, working overtime is essentially a job stressor having a clear association with adverse mental health [37]. The physiological recovery theory states that recovery activities, such as sleeping, exercising, or eating, are greatly nullified by working overtime, resulting in less time to relax or efficiently recover [38, 39]. Insufficient recovery can disturb physiological processes (blood pressure, hormone excretion, and sympathetic nervous system activity), eventually leading to psychological and physical health issues [40].

The results of our study are consistent with those of previous studies and theoretical inferences. Kleppa reported that overtime workers of both sexes have significantly higher levels of anxiety and depression and a higher prevalence of anxiety and depressive disorders than those working for normal hours [37]. In a cross-sectional study, Nishikitani found that working overtime was associated with increased Hamilton Depression Scale scores using univariate analysis [41], and Proctor determined that extended hours were associated with higher levels of depression [42]. Tyssen focused on suicidal tendencies, which can be considered as a severe depression-related outcome, and reported that longer working hours were associated with suicidal tendencies [43].

Insomnia has conventionally been conceptualized as a symptom of psychopathology, particularly depression [44]. More recently, insomnia has been considered a primary disorder if it is present without other clinically relevant psychiatric or medical diseases; otherwise, it was considered a secondary disorder [23]. Nevertheless, with respect to the association with depression, chronic insomnia can exist for years before the first onset of a depressive episode.

Our results suggested that insomnia is independently associated with DS among internet company employees. As proposed by Vandekerckhove, adequate sleep restores vitality for each working day, whereas employees with insufficient sleep were more sensitive to negative emotions and stressful events at work [45]. Moreover, daytime sleepiness resulting from insomnia can undermine attention, reactivity, and efficiency at work. Sleep dysfunction is reportedly associated with depersonalization toward learning among medical students, indicating that sleep disturbances reduce personal motivation at work. This reduced motivation ultimately promotes diffidence to academic learning and interferes with cognitive functions and self-assessment, resulting in mood dysfunction, such as depression [46]. In our study population, insomnia may lessen the ability of internet employees to tolerate the stressful work environment by interfering with sleep quality and quantity. Therefore, the results of the present study suggest that insomnia risks the mental health of those working in stressful environments such as internet companies.

Furthermore, a significant interaction effect of long working hours and insomnia on DS has been detected. Our results showed that the impact of working hours on DS varies between participants with or without insomnia and suggest that compared with the employees getting good sleep, those who experience insomnia and with long working hours have little chance to recover at night. Additional attention should be paid to employees with poor sleep quality when WT gets extended. On the other hand, working overtime shows more impact on the rate of DS in participants with insomnia than in those without insomnia. Further, a dose–response relationship appeared to exist between working hours and DS insomnia condition, considering the potential that along with the extension of working hours, employees with insomnia experience prolonged fatigue and frustration, thereby increasing the morbidity of DS.

This is the first study to investigate the independent and interaction effect of long working hours and insomnia on DS among internet company employees in China. The working hours followed in China are among the longest in the world; however, correlational research on long working hours remains limited.

There are some study limitations. First, the restricted type of occupation limited the extrapolation of conclusions to other professions. Moreover, the cross-sectional design cannot lead to any causal conclusion, which should be clarified in future prospective studies. Second, information was collected using self-administered questionnaires, which may lead to an increased risk of data unreliability. Furthermore, WT and insomnia were evaluated using a retrospective questionnaire, and recall bias could have influenced the interpretation of our exploratory findings.

## Conclusion

In this exploratory study with 3589 full-time employees from 35 internet companies, long working hours and insomnia are independent risk factors associated with DS. Moreover, interaction effects between WT and insomnia on DS were detected. The results suggest that to relieve DS, reducing insomnia is important, particularly among employees working for excessive hours at internet companies. Nevertheless, public policies promoting the reduction of working hours are warranted. Sometimes, long working hours may be inevitable; workers and enterprises should be informed of the potential risk and interventions that might mitigate the risk.

## Supplementary Information

**Additional file 1: Supplementary file 1.** The Sleep Questionnaire (睡眠问卷)

## Data Availability

Original data are available on reasonable request. These were stored on password-protected computers at the National Institute of Occupational Health and Poison Control, Chinese Center for Disease Control and Prevention, Beijing, China. Readers who wish to gain access to the data can write to the corresponding author.

## Abbreviations

DS: Depressive symptoms
PHQ-9: The patient health questionnaire
ORs: Odds ratios
DIS: Difficulty initiating sleep
DMS: Difficulty maintaining sleep
EMA: Early morning awakening
WT: Work time
CI: Confidence interval
