# Supplementary material for: Effect of long working hours and insomnia on depressive symptoms among employees of Chinese internet companies
Source: BMC Public Health. 2021 Jul 16;21:1408. doi: 10.1186/s12889-021-11454-9 (PMC8283851; doi:10.1186/s12889-021-11454-9)
Supplement: Supplementary file 1 — Additional file 1: Supplementary file 1. The Sleep Questionnaire (睡眠问卷) [file 12889_2021_11454_MOESM1_ESM.docx]

**Supplementary file 1**

**The Sleep Questionnaire（睡眠问卷）**

| Please answer according to your sleep status in the past year.  请根据您过去1年的睡眠情况作出回答。 |
| --- |
| 1. How long does it usually take you to fall asleep in bed?  您通常上床准备睡觉后多长时间能入睡？ |
| (1) 0–10 min 0~10分钟  (2) 11–30 min 11~30分钟  (3) 31–59 min 31~59分钟  (4) 1–2 h 1~2小时  (5) 2+ h 2小时以上 |
| 2. How often do you have difficulty staying asleep?  您是否夜里易醒，难以入睡？ |
| (1) Never (or almost never) 从来没有  (2) few times a year 一年数次  (3) more than once a month 每月1次以上  (4) more than once a week 每周1次以上  (5) more than 3 times a week 每周3次以上 |
| 3. How often do you wake up too early and cannot fall asleep again?  您是否经常醒来太早又不能再次入睡？ |
| (1) Never (or almost never) 从来没有  (2) few times a year 一年数次  (3) more than once a month 每月1次以上  (4) more than once a week 每周1次以上  (5) more than 3 times a week 每周3次以上 |
